# Impending role of inflammatory markers and their specificity and sensitivity in breast cancer patients

**DOI:** 10.1038/s41598-024-65821-8

**Published:** 2024-07-02

**Authors:** Samina Malik, Sulayman Waquar, Nimra Idrees, Arif Malik

**Affiliations:** 1 Department of Physiology, University College of Medicine and Dentistry (UCMD), The University of Lahore (UOL), Lahore, Pakistan; 2 Institute of Molecular Biology and Biotechnology (IMBB), UOL, Lahore, Pakistan; 3 Faculty of Health Sciences, Equator University of Science and Technology (EQUSaT), Masaka, Uganda

**Keywords:** Breast cancer, MMPs, ILs, HSPs, VEGF, Bcl2, Biochemistry, Cancer, Cell biology, Genetics, Molecular biology, Physiology

## Abstract

Cancer and related disorders are the most common cause of cancer-related mortality with the incidence of 1 in 9 among the pre-menopausal Pakistani females. among the most common ailments worldwide, indicating the importance of developing particular techniques that could help attenuate the effects of breast cancer and related outcomes. The primary aim of the current study was to review the role of inflammatory and stress markers in the development and progression of breast cancer. Four hundred ninety-eight (n = 498) patients with breast cancer and four hundred and ninety-eight (n = 498) age- and sex-matched controls were selected for this case‒control study. Serum samples were obtained, and the levels of stress and inflammatory markers, including Matrix metalloproteases (MMPs), Interleukins (ILs), Heat shock proteins (HSPs), Malondialdehyde (MDA), Nitric Oxide (NO), inducible Nitric Oxide Synthase (iNOS) and Tumour necrosis factor-alpha (TNF-α), were determined. Most (62%) patients had metastatic breast cancer (stage III or IV) with an adverse grade (65% with Grade III and 35% with Grade II). The present study showed that the levels of oxidants such as MDA, ILs, MMPs and HSPs were significantly greater, while the levels of antioxidants such as Superoxide Dismutase (SOD), Glutathione (GSH), Catalase (CAT), vitamin A, C and D were significantly lower in breast cancer patients than in controls, suggesting their diagnostic importance and role in the pathophysiology of breast cancer. Oxidants, including IL-1, HSP27 and MMP9, which are highly specific and sensitive, may be used to develop the pathophysiological pathways of metastatic breast cancer in these patients. These pathways include cell invasion, cell migration and epithelial–mesenchymal transition. Therefore, we concluded that an increase in growth factors, e.g., Vascular Endothelial Growth Factor (VEGF), Tumour Growth Factor-beta (TGF-β) and B-cell lymphoma (Bcl2), under the influence of these variables plays a crucial role in the metastasis of breast cancer.

## Introduction

Breast cancer remains as one of the most common multifactorial diseases in women worldwide. This disease is the major cause of mortality in both developed and developing countries^1^. Globally, eight million deaths were reported due to breast cancer in 2008, and this figure is estimated to increase to 11 million at the end of 2030^2^. Breast cancer is a metastatic cancer that can spread to other organs, such as the bone, liver, lungs, and brain, resulting in injury. Early detection of this disease can lead to improved prognosis and increased survival^3^. Early diagnosis of breast cancer using image processing techniques has been reported in the literature, major therapeutic goals include increasing life expectancy and controlling low-dose toxicity related to maintaining or improving quality of life^4^. There are two hypothetical theories in breast cancer research: the cancer stem cell theory and the stochastic theory^5^.

Risk factors for breast cancer include sex, age, race, family history and genetics, personal health history such as early and late menstruation, reproductive history, genetic mutations, congested breast tissue, malnutrition, obesity, lack of awareness, excessive alcohol consumption, radiation and nulliparous status, improper breastfeeding, oral contraceptives, delayed marriage and lifestyle^6–8^. Most patients with breast cancer have a better chance of survival if diagnosed early and treated appropriately^9,10^. Therefore, early detection and effective treatment are highly important for reducing the incidence and mortality of breast cancer. Breast self-examination (BSE) and mammography are believed to be the most effective ways to ensure early detection of breast cancer. Information and awareness of risk factors and the practice of testing, such as BSE, are important for reducing the incidence of breast cancer^11^. Chronic inflammation and involvement of inflammatory markers play significant role in onset and progression of breast cancer^12^. Inflammatory markers, such as C-reactive protein (CRP), interleukin-6 (IL-6), and tumour necrosis factor-α (TNF-α), have been shown to increase dramatically in response to infection, tissue damage, and active disease^13^. Differences in the degree of reference can predict the onset of health events, such as heart disease and disability, in people with no apparent inflammation^14^. Both the risk of cancer and plasma levels of inflammation increase with age, but it is not clear whether the variability in circulating marker levels is associated with an increased risk of cancer^15^. Research on rodents has provided direct evidence that genetic abnormalities and obesity increase inflammation in mammary gland cells, cytokine production, and morphological changes^16^. MMP-1 inhibited the invasive ability of various MDA-MB 231 cells in vitro and inhibited the growth of breast cancer cells when the cells were injected into rats. A reduction in MMP-1 expression significantly reduced brain metastasis and lung metastasis formation following cell injection into the left ventricle of the heart and tail, respectively^17^. Tumour necrosis factor-α (TNF-α) is an important inflammatory cytokine found in the TME that is involved in all stages of breast cancer growth, affecting cell proliferation and survival, epithelial-to-mesenchymal transition (EMT), metastasis and recurrence. In breast cancer, TGF-β has been suggested to play dual roles^18^. This molecule acts as a tumour suppressor in the early stages of the disease when it prevents the outflow of carcinomas in situ through its anti-inflammatory functions. In the later stages of this disease, TGF-β is believed to promote tumour progression, in part by improving cell motility and invasiveness and the ability to metastasize^19^. A tumour that promotes TGF-β expression correlates with the activity and increased protection of TGF-β by cancer cells during tumour progression. Interleukins determine the development, proliferation, and migration of breast cancer cells, affecting the onset of aggressive disease, angiogenesis and tumour growth, and are predictable factors that determine breast cancer survival rates^20^. Oestrogen stimulates the growth of oestrogen-positive breast cancer. This issue is why menopausal hormone therapy is contraindicated in women with breast cancer or a history of breast cancer to avoid increasing women's risk of breast cancer recurrence^21^.

## Materials and methods

Four hundred ninety-eight (n = 498) patients with confirmed breast cancer and four hundred ninety-eight (n = 498) healthy age- and sex-matched controls were recruited for this case‒control study. The diagnosis of breast cancer was confirmed via histopathology. All patients provided informed consent, the study was approved by the Ethical Review Committee of the Institute of Molecular Biology and Biotechnology, University of Lahore, Lahore, Pakistan and all of the methodology was performed in accordance with the relevant guidelines and regulations. Five millilitres of blood was drawn from the antecubital vein, and the serum was separated and stored at − 80 °C in a vial for future analysis before the patient started chemotherapy.

### Study design

Case–Control Study.

### Setting

Patients were selected from Inmol Cancer Hospital Lahore after getting their initial assessment if they met the selection criteria of the study during the interval of 2017–2020. All of the clinical and biochemical assessments were carried out at Institute of Molecular Biology and Biotechnology, The University of Lahore.

### Inclusion criteria

Females with clinically confirmed diagnosis of Breast Cancer age ranging from 35 to 65 years old, with at least one year of similar medical history.

### Exclusion criteria

Females with any other contagious and non-contagious disease were ruled out from the current study. And all the patients with improper medical record were excluded from the current study.

### Biochemical analysis

#### Determination of MMPS, ILs, HSPz, TNF-α, iNOS, and vitamin D

The levels of MMPs, ILs, HSPs, TNF-α, iNOS and vitamin D were determined with commercially available ELISA kits (Cayman Chemicals, USA).

#### Estimation of superoxide dismutase (SOD)

Superoxide dismutase (SOD) activity was predetermined according to the Kakkar procedure^22^. One hundred microlitres of sample was taken in a tube, and 1.2 ml of PBS, 100 μl of phenazine methosulfate, 300 μl of NBT and 200 μl of NADH were added. Thereafter, 100 μl of glacial acetic acid and 4 ml of 2-propanol were added to the tube, which was subsequently centrifuged (3000 rpm, 10 min). The absorbance was measured at 560 nm.

#### Determination of glutathione (GSH)

Glutathione levels were estimated according to the protocol of Moron et al. (1979)^23^. Then, 100 μl of serum was added to 0.02 M (2.4 μl) EDTA and ice-cooled (10 min), followed by the addition of 2 ml of distilled water. To this mixture, 50.0 μl of TCA (50%) was added, and the mixture was incubated on ice (10–15 min). The samples were then centrifuged (3500 rpm). The supernatant was removed, and 2 ml of 0.15 M Tris was added. HCl and 0.05 ml of DTNB were used. The absorbance was measured at 412 nm.

#### Determination of malondialdehyde (MDA)

This method was performed as described by Ohkawa et al.^24^. Two hundred microlitres of sample was added to the tube, to which 200 μl of 8.1% SDS and 1.5 ml of 20% acetic acid were added. Later, 1.5 ml of 0.8% TBA and 600 μl of distilled water were supplemented with 4 ml of 2-propanol. The mixture was centrifuged (4000 rpm, 10 min), and the supernatant was removed. Afterwards, the absorbance was measured at 532 nm using a UV-1100 spectrophotometer.

#### Determination of catalase (CAT)

Catalase (CAT) activity was determined by the method of Aebi (1974)^25^. One hundred microlitres of sample was added to the tube, followed by the addition of 1.9 ml of phosphate buffer and 1 ml of H_2_O_2_. Finally, three absorbance bands were measured every minute at a wavelength of 240 nm.

#### Estimation of nitric oxide (NO)

The Theories and Bories method was employed for this purpose^26^. A total of 100 μl of Griess Reagent was added to 300 μl of sample, which was subsequently supplemented with 2.6 ml of distilled water and incubated (30 min). The absorbance was measured at 548 nm.

#### Determination of vitamin A

The method described by Salvaraj and Susheela^27^ is a simple spectrofluorometric method for the detection of vitamin A in 50 μl serum samples. Assays of serum samples were performed at the microlitre level by the fluorometric technique and by the standard spectrophotometric technique at the millilitre level.

#### Determination of vitamin C

The vitamin C method of Chinoy et al. was used for estimation^28^. One hundred microlitres of the sample was added to 400 μl of 5% TCA and centrifuged (3000 rpm, 10 min). A total of 320 μl of supernatant was separated, 130 μl of DTC was added, and the mixture was allowed to warm (90 °C, 1 h). The mixture was subsequently ice-cooled, 600 μl of sulfuric acid was added, and the absorbance was subsequently measured at 520 nm.

### Statistical analysis

For the statistical analysis of the current study Independent T tests and Multivariate ANOVA (MANOVA) were performed on the data obtained in the following study using SPSS version 21. The results of the study are expressed as the Mean ± S.D., where p < 0.05 indicates significant findings. Moreover, contingency tables were constructed to determine the sensitivity, specificity, NPV, PPV and odds ratio (OR) among the selected breast cancer patients.

## Results

The data presented in Table 1, show that the majority (n = 287; 57.630%) of the patients with breast cancer in the current study were underweight compared with those with a normal weight (n = 122; 24.497%) or overweight/obese (n = 89; 17.871%). Most (n = 297; 59.638%) of these patients had occupations that did not strictly require mental (n = 88; 17.995%) or manual (n = 113; 22.690%) work. The educational status of most (n = 301; 60.441%) patients was primary school, whereas the remaining (n = 197; 39.558%) were high school or above. A lack of breastfeeding was observed in the majority (n = 391; 78.514%) of the patients compared with the controls (n = 107; 21.485%). A family history of breast cancer was negative for most (n = 411; 82.530%) patients but was present or some (n = 87; 17.469%) patients. This finding may indicate the dominance of recessive alleles due to possible parental consanguinity. Most (n = 401; 80.522%) breast cancer patients had a history of hormonal contraceptive use (n = 97; 19.477%, no contraceptive use), especially in association with low parity.

**Table 1:** Demographic and epidemiological parameters of breast cancer cases versus controls.

Variables	Cases (n = 498)	Cases (%)	Controls (n = 498)	Controls (%)
BMI				
Underweight	89	17.871	55	11.044
Normal weight	122	24.497	341	68.473
Overweight and obese	287	57.630	102	20.481
Occupation				
Manual worker	113	22.690	205	41.164
Mental worker	88	17.995	145	29.116
Others	297	59.638	148	29.718
Education				
Uneducated	301	60.44	265	53.212
Educated	197	39.55	233	46.78
Age (years)				
Mean age	36.50	–	35.34	–
Premenopausal	401	80.522	433	86.947
Postmenopausal	97	19.477	65	13.052
Age at first pregnancy				
21 years and below	149	29.919	112	22.489
22–29 years	233	46.787	256	51.405
30 and above	166	33.333	130	26.104
Parity				
Nulliparous	91	18.273	51	10.240
1–4	321	64.457	395	79.317
5 and above	86	17.269	52	10.441
History of breast feeding				
None	83	16.666	33	6.626
6 M & below	294	59.036	271	54.417
> 6 M to 1 year	47	09.437	55	11.044
> 1 year	74	14.859	139	27.911
Family history of breast cancer				
No	411	82.530	309	82.530
Yes	87	17.469	189	38.650
Parental consanguinity				
Yes	153	30.722	171	34.337
No	345	69.277	327	65.662
Contraceptive use (6 months & above)				
Yes	401	80.522	222	44.578
No	97	19.477	276	55.421
Systemic disease				
Cardiac disease	56	11.244	13	2.658
Liver disease	49	9.839	21	4.216
Lung/respiratory disease	120	24.09	42	8.43
Neurologic disease	22	4.417	26	5.220
No	251	50.401	396	79.518

*Underweight, if BMI =  < 18.5. Normal weight, if BMI = 18.5–24.9. Overweight and Obese, if BMI =  > 24.9

As shown in Table 2, left unilateral breast cancer was more common (n = 377; 75.702%) than right unilateral breast cancer was (n = 121; 24.297%). This finding may be associated with our preference for eating and drinking using the right hand. Likewise, right-sided breastfeeding may be preferred by predominantly right-handed mothers, who find it easier to feed from the right side most of the time holding the baby with their stronger arm. This phenomenon could result in the retention of secretions, resulting in chronic infection and carcinoma in the left breast. The patients were mostly ER-negative (n = 291; 58.433%), PR-positive (n = 341; 68.473%) and HER2-negative (n = 387; 77.710%). On TNM staging, most (n = 308; 61.847%) patients had metastatic (stage III and IV) breast cancer compared with those (n = 102; 20.481%) with stage II and n = 88; 17.670% with stage I. Invasive ductal carcinoma was the most prominent (n = 304; 61.044%) histopathological type among breast cancer patients. The remaining patients had invasive lobular carcinoma (n = 85; 17.068%), medullary carcinoma (n-58; 11.646%), papillary carcinoma (n = 19; 3.815%) or malignant phyllodes (n = 32; 6.425%). Most (n = 251; 50.401%) of the breast cancer patients had no comorbidities.

**Table 2:** Clinical parameters of breast cancer cases.

	Cases (n = 498)	Cases (%)
Breast involved		
Left	377	75.702
Right	121	24.297
ER status		
ER +	423	84.939
ER−	75	15.060
PR status		
PR +	431	86.546
PR−	67	13.453
HER2 status		
HER2 +	434	87.148
HER2−	64	12.851
TNBC		
Yes	209	41.967
No	289	58.032
TNM staging		
I	88	17.670
II	102	20.481
III and IV	308	61.847
Tumor grade		
Grade I	0.00	0.0000
Grade II	176	35.341
Grade III	322	64.658
Type of tumor		
Invasive ductal carcinoma	304	61.044
Invasive lobular carcinoma	85	17.068
Medullary carcinoma	58	11.646
Malignant phyllodes	32	6.425
Papillary carcinoma	19	3.815

*PR* progesterone receptor, *HER2* human epidermal growth factor receptor 2, *TNM* is tumor node and metastasis (grading system).

*ER* estrogen receptor.

The data in Table 3 reveal the role of oxidative stress and inflammatory markers, such as MDA, ILs, MMPs, and TNF-α, in the development and progression of breast cancer. Furthermore, the table indicates that the protective effects of various antioxidants and vitamins were significantly greater in the controls than in the patients. The results shown in Tables 3 and 4 highlight the pivotal role of HSP-27, MMP-9, IL-1, 4, 6, 8, NO and TNF-α in cell proliferation and metastasis. A contingency table was used to determine the sensitivity, specificity, and odds ratios for the variables among the patients with prevailing breast tumours as shown in Table 4.
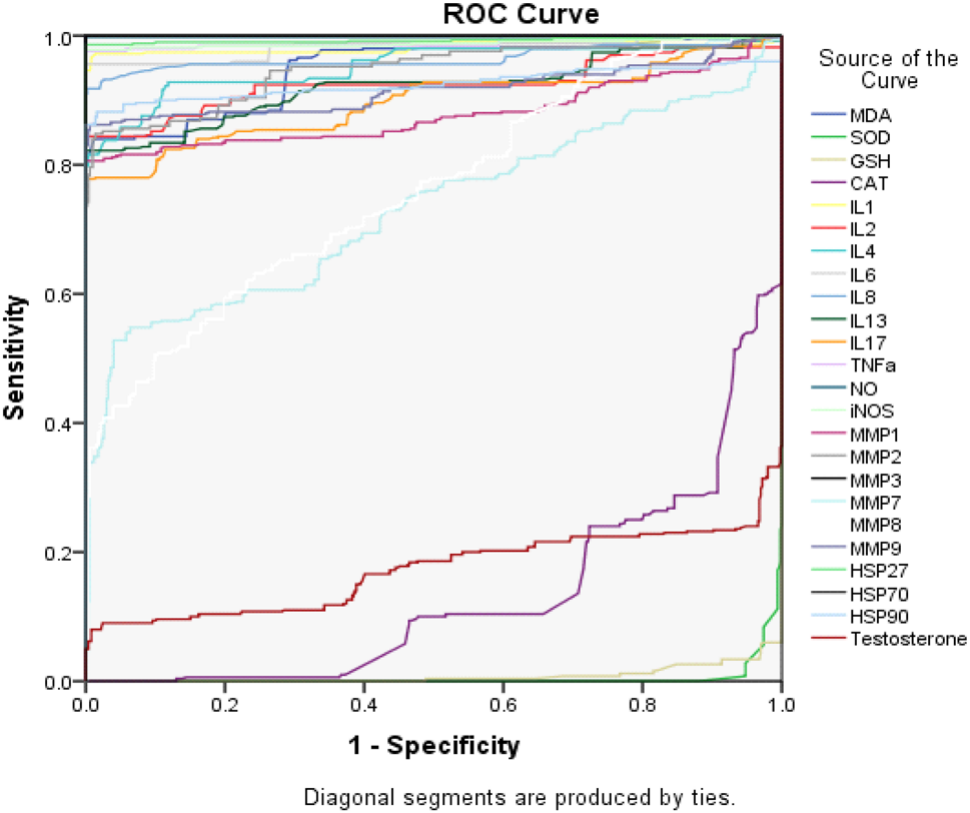

**Table 3:** Determination of oxidative and inflammatory markers within the cases.

Variable	Control (n = 498)	Patient (n = 498)	P Value
MDA nmol/ml	1.92 ± 0.059	3.77 ± 0.87	0.009
SOD µg/ml	0.54 ± 0.08	0.11 ± 0.01	0.013
GSH µg/ml	10.68 ± 1.23	4.42 ± 0.15	0.021
CAT nmol.mol	3.95 ± 0.11	2.13 ± 0.13	0.03
IL1 pg/ml	2.34 ± 0.53	6.97 ± 1.54	0.005
IL2 pg/ml	3.85 ± 0.64	7.84 ± 1.14	0.015
IL4 pg/ml	3.21 ± 0.70	7.44 ± 1.65	0.003
IL6 pg/ml	2.92 ± 0.69	8.81 ± 1.51	0.004
IL8 pg/ml	2.48 ± 0.56	6.09 ± 0.87	0.003
IL13 pg/ml	3.86 ± 0.74	7.45 ± 1.32	0.034
IL17 pg/ml	4.33 ± 0.95	8.71 ± 1.25	0.042
HSP27 pg/ml	4.41 ± 0.96	16.35 ± 1.37	0.003
HSP70 pg/ml	6.55 ± 1.38	22.81 ± 3.12	0.004
HSP90 pg/ml	6.05 ± 1.33	47.68 ± 4.87	0.003
TNFa pg/ml	13.13 ± 2.66	31.04 ± 8.64	0.012
NO ppm	5.95 ± 1.28	20.82 ± 4.08	0.019
iNOS ppm	5.45 ± 0.99	21.47 ± 8.76	0.005
MMP1 ng/ml	27.16 ± 4.50	55.83 ± 7.41	0.005
MMP2 ng/ml	37.50 ± 5.81	215.34 ± 16.49	0.039
MMP3 ng/ml	37.00 ± 6.06	204.81 ± 22.45	0.026
MMP7 ng/ml	45.26 ± 7.45	73.12 ± 8.73	0.031
MMP8 ng/ml	48.02 ± 8.15	76.71 ± 9.01	0.005
MMP9 ng/ml	42.00 ± 11.98	163.85 ± 22.38	0.015
Vitamin A IU	525.35 ± 81.92	406.23 ± 77.49	0.032
Vitamin C mg/dL	0.61 ± 0.01	0.36 ± 0.078	0.011
Vitamin D nmol/L	13.19 ± 0.91	9.46 ± 0.76	0.011

**Table 4:** Sensitivity, Specificity, NPV, PPV and odds ratio using 2 × 2 contingency tables.

Variables	SN %	SP %	NPV %	PPV %	Odds ratio (CI 95%)
MDA nmol/ml	80	62	14	98	6.73
SOD µg/ml	58	46	6	95	1.19
GSH µg/ml	48	54	5	95	1.09
CAT nmol.mol	55	52	6	96	1.36
IL1 pg/ml	82	79	19	99	17.43
IL2 pg/ml	68	64	10	97	3.79
IL4 pg/ml	77	75	14	98	9.51
IL6 pg/ml	72	72	12	98	6.67
IL8 pg/ml	60	60	7	97	2.29
IL13 pg/ml	62	58	8	97	2.33
IL17 pg/ml	61	66	8	97	3.10
HSP27 pg/ml	88	86	28	99	47.16
HSP70 pg/ml	76	72	14	98	8.48
HSP90 pg/ml	81	68	16	98	8.99
TNFa pg/ml	76	66	12	98	5.89
NO ppm	82	74	18	98	13.01
iNOS ppm	75	66	12	98	5.59
MMP1 ng/ml	58	45	5	95	1.12
MMP2 ng/ml	56	54	6	96	1.51
MMP3 ng/ml	57	60	7	96	1.99
MMP7 ng/ml	58	62	7	97	2.32
MMP8 ng/ml	72	54	9	97	3.15
MMP9 ng/ml	87	81	25	99	27.54
Vitamin A IU	60	58	7	96	2.10
Vitamin C mg/dL	75	44	8	96	2.26
Vitamin D nmol/L	64	48	7	96	1.65

Further, upon performing the multivariate analysis of the said inflammatory markers among the type of tumours niched in the current study, interesting relationships could be seen as shown in the Table 5. It shows how the levels of IL-1, 2, 4, 6, 17, MMP-8 and HSP-90 remained significantly different among one two types of tumours. The said significant difference highlights its role in the progression and development of certain tumour type and if they may be evaluated on the vast range would provide such evidences which would have their revolutionary role in understanding the disease pathology.

**Table 5:** Multivariate analysis of inflammatory markers among the different tumour types all of the reported values are at p < 0.05.

Dependent variable	(I) Type of tumour	(J) Type of tumour	Mean difference (I–J)	Std. error	95% CI	
					Lower bound	Upper bound
IL-1	Invasive ductal carcinoma	Medullary carcinoma	0.7668*	0.3639	0.0517	1.481
IL-2	Invasive ductal carcinoma	Papillary carcinoma	1.0842*	0.50673	0.0885	2.07
	Invasive lobular carcinoma	Papillary carcinoma	1.3470*	0.54377	0.2786	2.4154
IL-4	Invasive ductal carcinoma	Papillary carcinoma	1.3006*	0.625	0.0723	2.5288
IL-6	Medullary carcinoma	Papillary carcinoma	1.4263*	0.693	0.0631	2.7896
IL-17	Invasive lobular carcinoma	Papillary carcinoma	1.2714*	0.572	0.1456	2.3971
MMP-8	Invasive ductal carcinoma	Medullary carcinoma	− 138.5159*	49.85	− 236.47	− 40.56
	Invasive lobular carcinoma	Medullary carcinoma	− 138.7155*	59.25	− 255.14	− 22.28
HSP-90	Invasive lobular carcinoma	Medullary carcinoma	− 8.4724*	4.23	− 16.79	− 0.152

## Discussion

Matrix metalloproteases (MMPs) (and members of the ADAM protein family of related metalloproteinases) can activate epidermal growth factor (EGF) receptors through the release of cell-related ligands, such as HB-EGF, TGFβ, and amphiregulin^29^. Research over the past decade has revealed that MMPs can promote chemical resistance and tumour progression through proteolytic inactivation of the cell receptor Fas and subsequent inhibition of internal apoptosis; preventing the separation of Fas by MMPs is a therapeutic intervention^30^. MMPs can also affect the tumour microenvelope by promoting the development of activated stromal cells. Myofibroblasts are important sources of MMPs in breast cancer, and tumour progression and adverse reactions are associated with strong expression of MMP-1, MMP-7, and MMP-9, as well as fibroblast-specific production of MMP-9, MMP-11, and MMP-14^31^. Cancer cells can also directly release various collagen isoforms that are resistant to MMP fragmentation and can serve as indicators to direct cancer cell invasion in small areas rich in MMPs^32^. Another important source of MMPs in breast cancer is adipocytes, which are associated with tumours. Cellular genetic factors trigger the breakdown of adipocytes, which have more tissue around the developing breast cancer, resulting in a phenotype associated with an increase in cytokines and MMP-11, which attacks breast cancer and metastasis. MMPs have long been known to promote cancer cell attack by destroying the ECM, but MMPs can act directly on tumour cells to promote invasive cellular features^33^. MMP-9 regulates cancer cell proliferation and metastasis in part by clearing Wnt/planar cell polarity protein-tyrosine kinase-7 (PTK7). MMP-3 promotes spontaneous tumour formation in mice, and subsequent analysis of this process revealed that the exposure of cultured mammary epithelial cells to MMP-3 directly activated EMT. It is possible that many studies in which MMPs have been shown to promote cell cancer and cancer invasion, although not directly investigated in these cases in the context of EMT, actually examined the cellular effects of incomplete or damaged EMT systems^34^. MMPs can also act as signalling-independent molecules through their proteolytic activity. The interaction of MMP substrates with subdomains of non-MMPs is known to affect the selection of specific substrates and target areas within those substrates. The MMP activities and specifications have been described. These combinations may also further signal functions: interaction of MMP-2 or MMP-9 haemopexin domain-containing compounds or CD44 may promote cell survival, migration and angiogenesis^35^. Two novel functions of the MMP-3 haemopexin domain include interaction with extracellular heat shock protein 90-β (HSP90β), which may promote invasion of mammary epithelial cells and morphogenesis, while binding of the MMP-3 haemopexin domain to Wnt5b prevents canonical Wnt signalling and regulates the formation of mammary stem cells^36^. The interaction of proteins with subunits with different TIMP-1 subunits in the inhibitory MMP domain also contributes to tumour growth. The independent MMP interaction of TIMP-1 with CD63 results in apoptosis resistance, EMT implantation, and cell division-related stemness. The active inhibition of the protumorigenic activity of MMPs and TIMPs is needed to determine the fusion and noncatalytic activities of these molecules^37^. The activity of MMP-2 and MMP-9 in serum or plasma, measured by gelatine zymography, has been shown to distinguish between breast cancer subclassification and various risk factors, predict lymph node metastasis, and evaluate treatment response in breast cancer patients. Other studies have evaluated the concentration of the MMP-9 protein in serum by ELISAs or by Luminex multiplex protein assays to provide an effective alternative to the same predictive value, and a higher serum MMP-2 concentration measured by ELISAs is also associated with incorrect prediction. Both matrix degradation enzymes have antiapoptotic effects. MMPs and ADAM, especially MMP-7 and ADAM10, provide apoptotic signals to cancer cells by removing the Fas ligand, a transmembrane receptor Fas, on the cell surface. This proteolytic activity activates the Fas receptor and induces resistance to apoptosis and chemoresistance in cancer cells or promotes apoptosis in neighbouring cells according to the system. In addition, proteolytic depletion of phase-related tumour-protein-related protein I by ADAM17 may suppress the fatal cell-specific cytotoxicity of cancer cells. Notably, MMPs may contribute to the antiapoptotic effect by indirectly activating the serine/threonine kinase Akt/protein kinase B through the EGFR and IGFR cassettes. MMPs also promote apoptosis, possibly indirectly by altering the structure of the ECM, for example, by cleaving laminin, which influences the integrin signature.

The most common type of breast cancer, IDC, starts with growing cells and involves establishment of tubes, attacks on fatty tissue outside the canal and the development of a smooth and heavy tumour in the chest^38^. IL-1 expression was significantly greater in IDC breast tissue than in healthy tissue. IL-1 levels have been proven to be consistent with early tumour status, advanced tumour stage, high levels of lymph node emergence and distant metastasis, HER2 status, and the presence of mitotic counts^39^. High IL-1 expression levels predict higher survival rates and metastasis survival in patients with a risk greater than three times greater (HR = 3.322). However, the serum levels of IL-1 are not altered by the tumour phase, suggesting that IL-1 levels in a small local area are more important than the effect on the breast cancer system. Therefore, IL-1 expression in tumour cells is considered to be involved in foetal progression and is correlated with the clinical outcome of patients with breast cancer^40^. Therefore, the IL-1 concentration has the potential to be an indicator for patients with IDC breast tumours. IL-1 acts on autocrine tissue, and the expression and colocalization of IL-1 and its cognate receptors can be observed in both human (MCF-7 and Hs578T) and mouse (67NR and 4T1) breast cancer cell lines. In vitro studies have shown that IL-1 activation in 4T1 cells involves the intracellular signals STAT3, JNK, ERK, AKT, and NF-κB, which may be involved in cell proliferation and survival, but phosphorylation occurs only at the JNK, ERK, and AKT levels in Hs578T human breast cancer cells. Fibronectin (FN) expression and tumour cell interactions are well-known promoters of tumour progression and lung metastasis in patients with breast cancer. IL-1 treatment improves certain growth and migratory functions, as does fibronectin expression and binding to both human and rat breast cancer cells. In MCF-7 cells, IL-19 directly promotes proliferation, leading to an increase in the percentage of cells in the G2/M phase of the cell cycle^41^. Exposure to chronic fibronectin and gastric cell migration have been confirmed in IL-1, 2, 4, 17, and 19 knockdown 4T1 cells, while IL-1 treatment continues to increase migration in IL knockdown cells^42^. In addition, IL-1 overexpression promoted the proliferation, migration, tumour growth, and metastasis of breast cancer cells. CXCR4, one of the most prominent chemokine receptors secreted by breast cancer cells and attracted by its ligand, SDF-1cy, is essential for targeted breast cancer metastasis^43^. IL-1 reduces the expression of CXCR4 in breast cancer cells. An active hypoxic factor has been reported in the IL-1 promoter, while hypoxia reduces IL-1 expression in association with an increase in CXCR4 in breast cancer cell lines. After chemical response (CoCl2) and physiotherapy (hypoxia chamber) treatment for hypoxia, an anti-IL-1 mAb was used to reduce hypoxia induced by CXCR4 expression in vitro^44^. Therefore, the expression of IL-1 can increase in combination with hypoxia induced by CXCR4 and metastasis. T-cell activation and proliferation by releasing IL-10, TGF-β, and prostaglandins. Th2 cytokines promote M2-polarized TAM phenotypes and are very important for eliminating the immune system and inflammatory circuits that promote tumour growth and progression^45^. In breast carcinomas, a higher rate of TAM infiltration is associated with a worse prognosis. IL-19 converts Th1 and Th2 cells into Th2 cells. This molecule stimulates the secretion of Th2 cytokines (IL-4, -5, -10, and -13) and reduces Th1 cytokine depletion (IFN-γ)^46^. IL-17 treatment induces IL-19 expression, and the levels of IL-13 and -19 are closely related to the Th2 response to human asthma. Treatment of LPS-stimulated monocytes with IL-4 or IL-13 enhances IL-19 expression. There may be a positive response between IL-19 and Th2 cytokines^47^. Whether Th2 cytokines promote the function of TAMs has been confirmed; however, the relationship between IL-19 and T2-polarized TAMs remains to be determined. Interleukin (IL)-6 can play a key role in the growth and development of cancer cells, in the formation of osteolysis and humoral hypercalcaemia, and in the regulation of oestrogen production in breast cancer cells. IL-6 has also been suggested to be a cachectic factor in cancer patients. Decreased serum IL-6 levels induced by medroxyprogesterone acetate (MPA) have been reported to be associated with weight loss in patients with advanced breast cancer^48^. Elucidation of the mechanisms underlying the anticachectic effect of MPA showed that the secretion of IL-6 from the KPL-4 cell line, the first cell line of human breast cancer, induces cachexia. It has been suggested that the inhibitory effect of MPA on IL-6 secretion from breast cancer cells causes the anticachectic effect of MPA. Several of our studies have shown that 5'-fluorouridine (5'-DFUR) inhibits KPL-4 tissue growth and lowers IL-6 levels in both serum and tumour tissues^49^. Decreased serum IL-6 levels led to reduced weight loss. Docetaxel increased IL-6 levels in both serum and KPL-4 tissues, but combined treatment with docetaxel and 5'-DFUR not only led to a stronger antitumour effect but also caused a dramatic decrease in the serum IL level.

**Figure 1:**
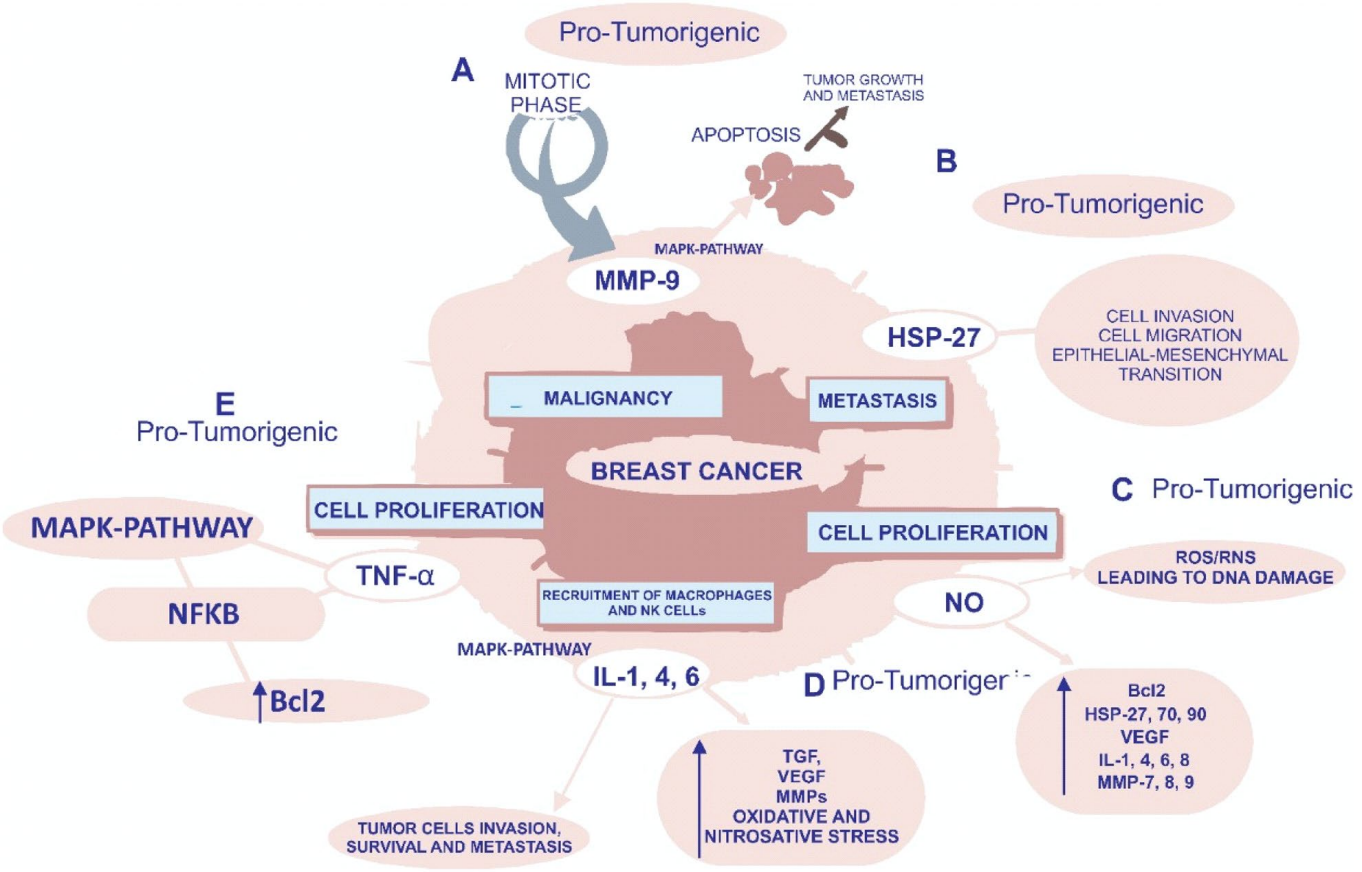
The postulated role of inflammatory pathways in the development and progression of breast cancer.

Hsp90 functions as a molecular regulator in cells by binding to a group of active proteins called cochaperones. Among these proteins, p50/Cdc37 has been shown to be highly expressed in most cancers and is needed for the maturation of a wide range of oncogenic protein kinases. Cdc37 has been tested for its ability to target cancer and appears to be very effective at least in prostate cancer^50^. Cdc37 has therapeutic potential due to its high potential for cancer screening and ongoing testing to identify breast cancer proteins^51^. Hsp70 appears to be effective at treating breast cancer due to its ability to prevent systemic cell death and senescence after exposure to high levels of Hsp70, which are important features of fatal mutations^52^. Hsp70 can therefore lead to cell activation by allowing planned cell death. However, unlike those available for Hsp90, effective drugs for targeting the ATP-binding domain of Hsp70 are currently unavailable. However, as Hsp70 inhibits apoptosis at the postmitochondrial level by activating the apoptosome and AIF, Hsp70-targeting techniques appear to be effective at overcoming tumour cell resistance. In breast cancer, Hsp27 has severe side effects due to its ability to cause structural anti-cell death and senescence^53^. Hsp27 is activated in both stress-induced enlargement and phosphorylation by p38 MAPK stress kinase, but it is unclear which of these processes are involved in Hsp27 upregulation in cancer. However, HSF1 functions in breast cancer, and Hsp27 levels may increase a second time during this change. After activation, Hsp27 regulates its cellular activity through a phosphorylation and oligomerization-based process involved in its protein function and cell threats. Unlike other HSPs, sHSPs do not bind to ATP, a substance that can cause this molecule to be difficult to identify with small chemicals. Many active anticancer agents target the ATP-binding domains of protein kinases or other cellular chaperones. As the central moderator of the HSP discourse, HSF1 may be a platform for innovative ways to treat breast cancer. HSF1 dysfunction has been shown to inhibit cancer progression^54^. In addition, the HER2/neu pathway has been shown to induce HSF1 and HSP expression and to induce autoimmune and drug-induced cell death. HSF1 can also mediate tumorigenic effects through another mechanism involving the recruitment of the prometastatic gene corepressor MTA1, which inhibits the expression of antimetastatic genes as shown in Fig. 1^55^. All of the said correlations could be seen in Table 6, MTA1 thus appears to act as a crossroads between oestrogen and Her2/neu regulatory signalling, promoting metastasis, and HSF1 appears to be involved in these effects. HSF1 also has additional functions in cancer, including the development of dangerous signatures through the use of ERK, PKA and TOR^56^.

**Table 6:** PEARSON’S correlation coefficients matrix among the potential variables of breast cancer pateints.

	MDA	SOD	GSH	CAT	VIT-C	VIT-D	IL1	IL2	IL4	IL6	IL8	IL13	IL17	TNFa	NO	iNOS	MMP1	MMP2	MMP3	MMP7	MMP8	MMP9	HSP27	HSP70	HSP90
MDA	1	− 0.801**	− 0.940**	− 0.933**	0.008	0.089*	0.939**	0.499**	0.963**	0.944**	0.371**	0.474**	0.491**	0.963**	0.949**	0.390**	0.924**	0.929**	0.912**	0.902**	0.219**	0.849**	0.924**	0.962**	0.417**
SOD		1	0.907**	0.905**	− 0.002	− 0.092*	− 0.855**	− 0.394**	− 0.850**	− 0.931**	− 0.164**	− 0.354**	− 0.388**	− 0.791**	− 0.851**	− 0.400**	− 0.923**	− 0.829**	− 0.944**	− 0.921**	− 0.084	− 0.618**	− 0.874**	− 0.811**	− 0.343**
GSH			1	0.962**	− 0.017	− 0.108*	− 0.948**	− 0.477**	− 0.956**	− 0.977**	− 0.321**	− 0.467**	− 0.484**	− 0.948**	− 0.957**	− 0.420**	− 0.962**	− 0.943**	− 0.969**	− 0.954**	− 0.139**	− 0.819**	− 0.951**	− 0.942**	− 0.400**
CAT				1	− 0.019	− 0.098*	− 0.971**	− 0.508**	− 0.951**	− 0.975**	− 0.316**	− 0.436**	− 0.482**	− 0.949**	− 0.963**	− 0.466**	− 0.979**	− 0.968**	− 0.971**	− 0.965**	− 0.122**	− 0.832**	− 0.973**	− 0.961**	− 0.461**
VIT-C					1	0.005	0.015	0.023	0.002	0.012	− 0.025	0.031	0.014	0.020	0.004	0.000	0.012	0.014	0.008	0.011	0.000	0.035	0.008	0.011	− 0.024
VIT-D						1	0.101*	0.092*	0.078	0.098*	0.054	0.057	0.048	0.107*	0.092*	0.105*	0.095*	0.099*	0.100*	0.093*	− 0.024	0.122**	0.101*	0.097*	0.054
IL1							1	0.507**	0.967**	0.961**	0.353**	0.432**	0.497**	0.975**	0.979**	0.479**	0.982**	0.987**	0.956**	0.978**	0.121**	0.862**	0.986**	0.984**	0.497**
IL2								1	0.490**	0.487**	0.319**	0.368**	0.544**	0.518**	0.505**	0.241**	0.487**	0.510**	0.476**	0.475**	0.090*	0.510**	0.501**	0.522**	0.265**
IL4									1	0.972**	0.358**	0.470**	0.491**	0.960**	0.984**	0.433**	0.964**	0.960**	0.956**	0.958**	0.194**	0.822**	0.965**	0.973**	0.455**
IL6										1	0.317**	0.448**	0.481**	0.939**	0.970**	0.446**	0.984**	0.950**	0.990**	0.976**	0.173**	0.803**	0.970**	0.954**	0.434**
IL8											1	0.327**	0.405**	0.394**	0.372**	0.169**	0.325**	0.384**	0.311**	0.312**	0.071	0.432**	0.362**	0.400**	0.172**
IL13												1	0.463**	0.469**	0.462**	0.185**	0.431**	0.458**	0.440**	0.413**	0.101*	0.462**	0.445**	0.469**	0.173**
IL17													1	0.507**	0.501**	0.259**	0.487**	0.511**	0.477**	0.476**	0.072	0.477**	0.499**	0.512**	0.223**
TNFa														1	0.965**	0.436**	0.944**	0.977**	0.921**	0.931**	0.161**	0.919**	0.962**	0.986**	0.468**
NO															1	0.473**	0.978**	0.982**	0.967**	0.970**	0.141**	0.848**	0.989**	0.986**	0.475**
iNOS																1	0.472**	0.475**	0.461**	0.485**	0.031	0.393**	0.485**	0.461**	0.482**
MMP1																	1	0.973**	0.987**	0.993**	0.116**	0.809**	0.989**	0.965**	0.463**
MMP2																		1	0.949**	0.962**	0.125**	0.882**	0.991**	0.987**	0.487**
MMP3																			1	0.985**	0.123**	0.779**	0.976**	0.943**	0.436**
MMP7																				1	0.109*	0.777**	0.983**	0.953**	0.472**
MMP8																					1	0.125**	0.116**	0.170**	0.040
MMP9																						1	0.853**	0.892**	0.444**
HSP27																							1	0.982**	0.485**
HSP70																								1	0.484**
HSP90																									1

*Correlation is significant at the 0.05 level (2-tailed).

**Correlation is significant at the 0.01 level (2-tailed).

## Conclusion

The current study helps in determining the potential variables that may be involved in the development and progression of breast cancer among the population. Increased levels of oxidative and nitrosative stress species under the influence of increased levels of inflammatory cytokines lead to increased incidences of cell invasion, cell migration and epithelial–mesenchymal transition. Hence, upregulation of the inflammatory markers i.e., TNF-α, MMPs, HSPs and ILs have significant interplay in the onset and progression of breast cancer.

## Data Availability

The data that support the findings of this study can be obtained from the corresponding author upon reasonable request. Any additional information needed to reproduce this work can also be obtained from the corresponding author.
